# Supplementary material for: Inhibition of the metalloprotease ADAM19 as a novel senomorphic strategy to ameliorate gut permeability and senescence markers by modulating senescence-associated secretory phenotype (SASP)
Source: Aging (Albany NY). 2025 Mar 20;17(3):757–77. doi: 10.18632/aging.206224 (PMC11984429; doi:10.18632/aging.206224)
Supplement: Supplementary Figures [file aging-17-206224-s001.pdf]

SUPPLEMENTARY FIGURES

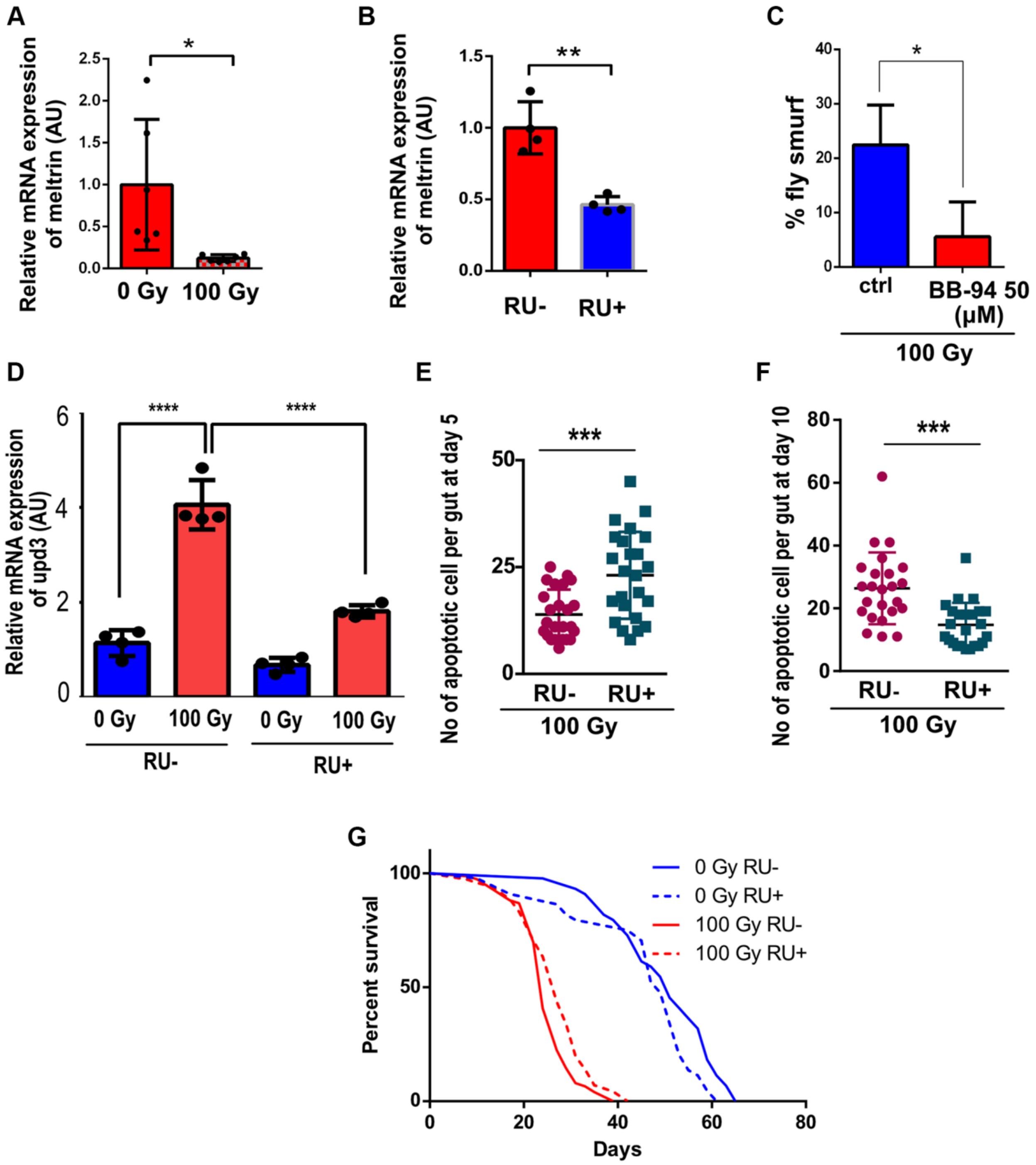

Supplementary Figure 1. (**A**) Relative mRNA expression of *meltrin* detected by qRT-PCR in flies upon X-Ray irradiation. (**B**) Relative mRNA expression of *meltrin* detected by qRT-PCR in the gut of *meltrin* RNAi flies. (**C**) % of Smurf-positive flies with leaky gut upon 50 μM BB-94 treatment after 100 Gy X-Ray exposure. (**D**) Relative mRNA expression of upd3 detected by qRT-PCR upon X-Ray irradiation and *meltrin* knockdown. (**E**) Quantification of apoptotic cells upon *meltrin* knockdown and 5-day post-X-ray irradiation. (**F**) Quantification of apoptotic cells upon *meltrin* knockdown and 10-day post-X-ray irradiation. (**G**) The lifespan of *Drosophila* in flies with X-ray exposure (100 Gy), No X-ray exposure (0 Gy), *meltrin* knockdown (RU+) in ECs, and no *meltrin* knockdown control (RU-). Error bars indicate SEM. The student's *t*-test determined the significance level by asterisks: $^{*}p < 0.05$, $^{**}p < 0.01$, $^{***}p < 0.001$, and $^{****}p < 0.0001$.

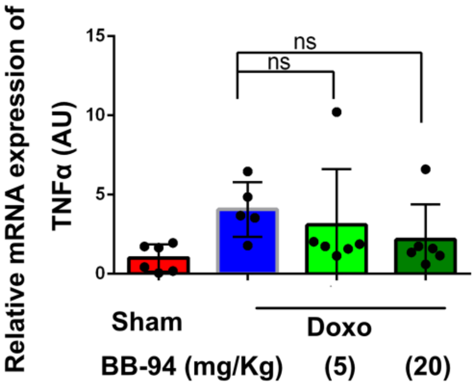

Supplementary Figure 2. Relative mRNA expression of TNFα in mouse colon tissues. Error bars indicate SEM. The significance level was determined by ANOVA and denoted by ns (no significance).

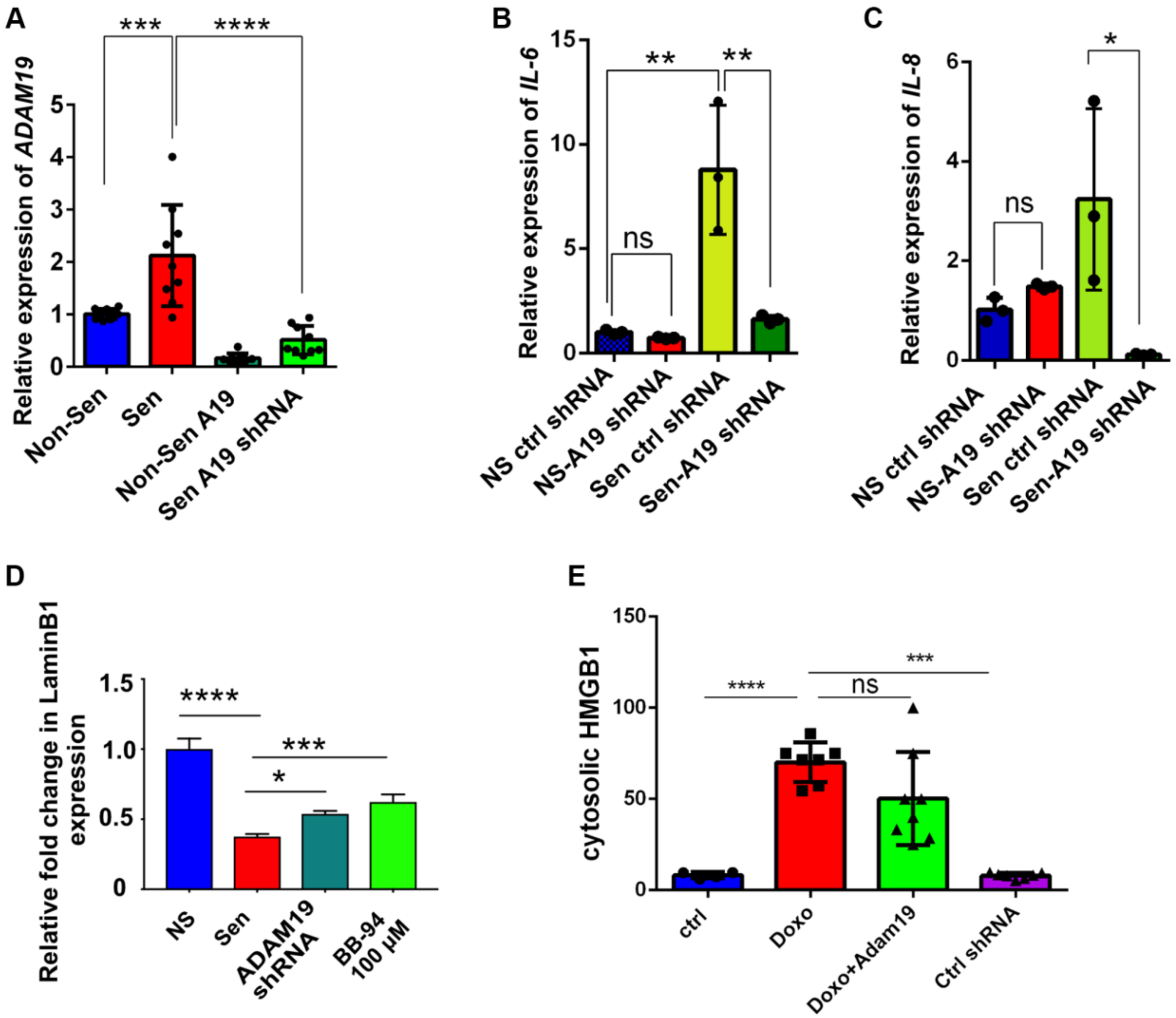

Supplementary Figure 3. (**A**) *ADAM19* expression upon senescence and non-senescence with ADAM 19 0r shRNA control treatments. (**B**) Relative mRNA expression of IL-6 in IMR90 cells upon senescence and non-senescence with ctrl shRNA and *ADAM19* shRNA transduction. (**C**) Relative mRNA expression of IL-8 in IMR90 cells upon senescence and non-senescence with ctrl shRNA and *ADAM19* shRNA transduction. (**D**) Lamin B expression in control, senescence, senescence with *ADAM19* shRNA expressing and with BB-94 treatment cells. (**E**) % cells count with cytosolic HMGB1 in control, senescence, and senescence with *ADAM19* shRNA expressing cells. Error bars indicate SEM. The significance level was determined by ANOVA and denoted by asterisks: $^{*}p < 0.05$, $^{**}p < 0.01$, $^{***}p < 0.001$, and $^{****}p < 0.0001$. In (**A**–**D**) “NS” represent non senescence and ‘Sen’ represent senescence.
